# Casticin inhibits nasopharyngeal carcinoma growth by targeting phosphoinositide 3-kinase

**DOI:** 10.1186/s12935-019-1069-6

**Published:** 2019-12-21

**Authors:** Jingxian Liu, Jinghong Yang, Yuhe Hou, Zhenwei Zhu, Jie He, Hao Zhao, Xidong Ye, Dengke Li, Zhaohui Wu, Zhongxi Huang, Bingtao Hao, Kaitai Yao

**Affiliations:** 1 Guangdong Provincial Key Laboratory of Tumor Immunotherapy, Cancer Research Institute, School of Basic Medical Sciences, Southern Medical University, Guangzhou, 510515 Guangdong People’s Republic of China; 2 Shenzhen Hospital, Southern Medical University, Shenzhen, 518000 Guangdong People’s Republic of China; 3 Shunde Hospital, Southern Medical University, Shunde, 528300 Guangdong People’s Republic of China

**Keywords:** Casticin, Nasopharyngeal carcinoma, Target, PI3K/AKT pathway

## Abstract

**Background:**

Casticin, an isoflavone compound extracted from the herb Fructus Viticis, has demonstrated anti-inflammatory and anticancer activities and properties. The aim of this study was to investigate the effects and mechanisms of casticin in nasopharyngeal carcinoma (NPC) cells and to determine its potential for targeted use as a medicine.

**Methods:**

NPC cells were used to perform the experiments. The CCK‑8 assay and colony formation assays were used to assess cell viability. Flow cytometry was used to measure the cell cycle and apoptosis analysis (annexin V/PI assay). A three-dimensional (3D) tumour sphere culture system was used to characterize the effect of casticin on NPC stem cells. In silico molecular docking prediction and high-throughput KINOME scan assays were used to evaluate the binding of casticin to phosphoinositide 3-kinase (PI3K), including wild-type and most of mutants variants. We also used the SelectScreen assay to detect the IC50 of ATP activity in the active site of the target kinase. Western blotting was used to evaluate the changes in key proteins involved cell cycle, apoptosis, stemness, and PI3K/protein kinase B (AKT) signalling. The effect of casticin treatment in vivo was determined by using a xenograft mouse model.

**Results:**

Our results indicate that casticin is a new and novel selective PI3K inhibitor that can significantly inhibit NPC proliferation and that it induces G2/GM arrest and apoptosis by upregulating Bax/BCL2 expression. Moreover, casticin was observed to affect the self-renewal ability of the nasopharyngeal carcinoma cell lines, and a combination of casticin with BYL719 was observed to induce a decrease in the level of the phosphorylation of mTORC1 downstream targets in BYL719-insensitive NPC cell lines.

**Conclusion:**

Casticin is a newly emerging selective PI3K inhibitor with potential for use as a targeted therapeutic treatment for nasopharyngeal carcinoma. Accordingly, casticin might represent a novel and effective agent against NPC and likely has high potential for combined use with pharmacological agents targeting PI3K/AKT.

## Background

Nasopharyngeal carcinoma is one of the most common cancers of the head and neck; it has an extremely skewed ethnic and geographic distribution and is highly prevalent in Southern China and Southeast Asia [1]. The main treatments strategies for patients with nasopharyngeal carcinoma are ionizing radiation and chemo-radiation therapy [2]. Although both treatment methods have revealed good results and outcomes, there still remains a substantial proportion of patients with nasopharyngeal carcinoma who do not respond to treatments designed to prevent disease recurrence and who die as a consequence of drug resistance and metastasis [3]. Therefore, finding increasingly efficient and effective targeted therapeutic strategies to treat nasopharyngeal carcinoma remains as an important task.

**Fig. 1:**
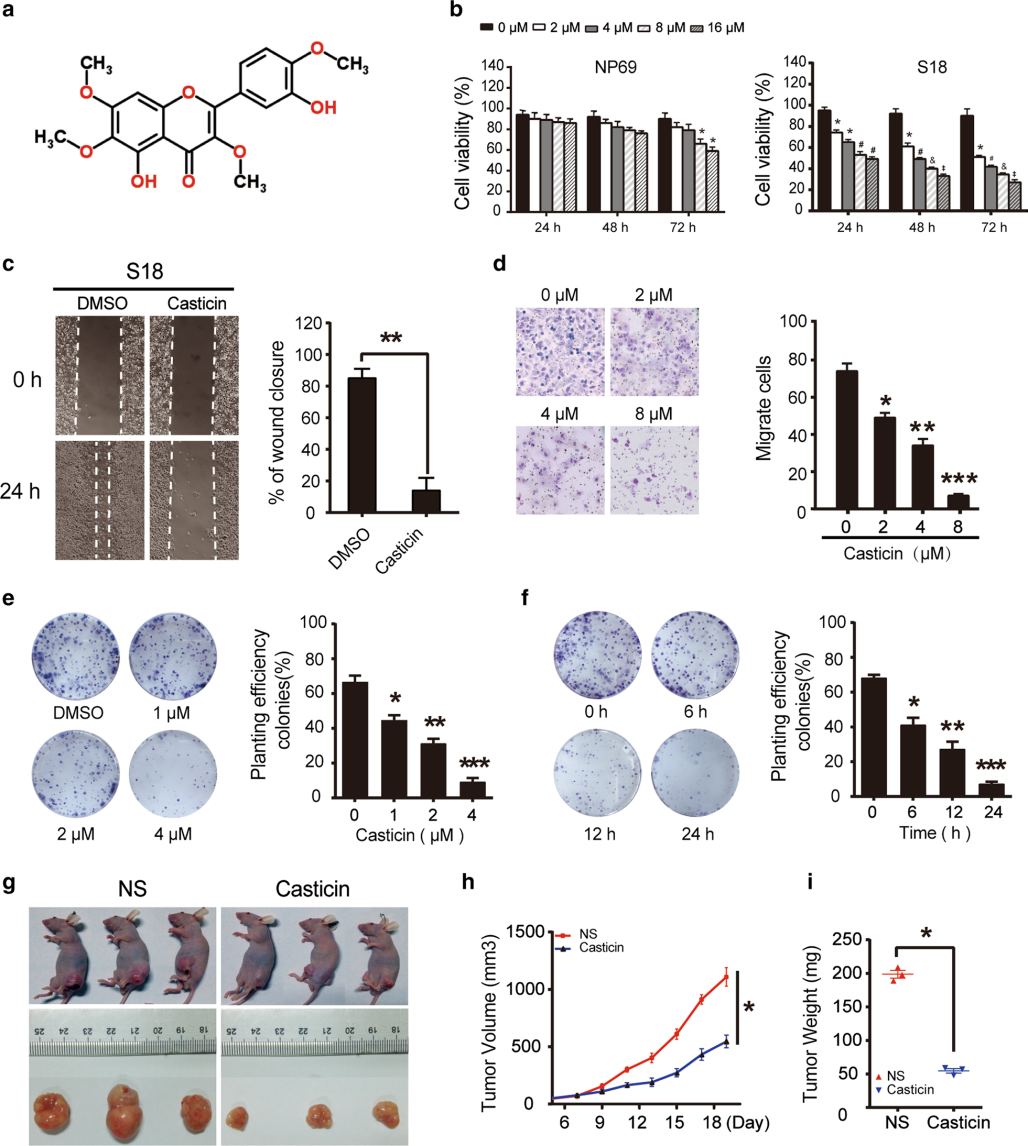
Casticin inhibits the proliferation and viability of NPC cells in vitro and in vivo. **a** The chemical structure of casticin. **b** NP69 and S18 cells were treated with a gradient of casticin concentrations (0, 2, 4, 8, 16 µM) for 24, 48 or 72 h. Cell viability was assessed using the CCK-8 assay. The data are presented as the mean ± SEM, *p < 0.05 versus 0 µM; ^**#**^*p* < 0.05 versus 2 µM; ^&^*p* < 0.05 versus 4 µM; ^‡^*p* < 0.05 versus 8 µM. **c** Casticin inhibits migration of S18 cells in the wound healing assay. White dashed lines indicate the wound edge. The residual gap between the migrating cells from the opposite edges of the wound is represented by a percentage of the initial scratch area. Corresponding graph shows the mean width of the injury lines of three independent experiments (right). All data are presented as the mean ± standard deviation. **p < 0.01 versus DMSO. **d** Casticin inhibits migration of S18 cells in the Transwell assay. Corresponding graphs in the panel on the right show the mean numbers of cells per high-power field (HPF) from five independent areas. All data are presented as the mean ± standard deviation. *p < 0.05 versus 0 µM, **p < 0.01 versus 2 µM, and ***p < 0.001 versus 4 µM. **e**, **f** Colony formation of S18 cells post-treatment with casticin. Cells were exposed to 0, 1, 2, and 4 µM casticin for 12 h or were exposed to a fixed level of 1 µM casticin for different time periods (0, 6, 12, and 24 h) and were then allowed to form colonies for approximately 10 days. Corresponding graphs show the mean number of colonies in different groups for three independent experiments (right). All data are presented as the mean ± standard deviation. Right panel of **e** *p < 0.05 versus 0 µM, **p < 0.01 versus 1 µM, and ***p < 0.001 versus 2 µM. Right panel of **f** *p < 0.05 versus 0 h, **p < 0.01 versus 6 h, and ***p < 0.001 versus 12 h; **g** Casticin (10% DMSO + 90% physiological saline, 40 mg/kg) was injected into nude mice every day for 12 days starting 6 days after inoculation with 5 million S18 cells. **h** Tumour volume was periodically measured for each mouse, and the growth curve was plotted. **i** Tumours were excised from the animals and weighed. *p < 0.05 versus NS

Casticin (3′,5-dihydroxy-3,4′,6,7-tetramethoxyflavone, Fig. 1a), also known as vitexicarpin, is one of the main active constituents of the Chinese traditional herb Fructus Viticis [4]. Many studies have reported that casticin can significantly inhibit the proliferation and migration of many types of cancer cells, including leukaemia cells and ovary, stomach, liver, lung, and colon cancer cells [4–6]. However, the roles and dynamics related to its ability to inhibit nasopharyngeal carcinoma have not been investigated. Previous studies have indicated that there are many mechanisms involved in the apoptosis of cancer cells in responseto casticin, including caspase-3 activation, G2/M phase arrest, activation of FoxO3a, activation of PI3K/Akt, etc. [7, 8]. However, the direct molecular targets of casticin are still unknown, which limits its clinical applications.

Phosphatidylinositol 3-kinases (PI3Ks), a class of lipid kinases, play important roles in the signal integration of cytokines, growth factors, and cellular environmental components [2, 9], which transform into intracellular signals that regulate a variety of signalling pathways. These pathways are responsible for a variety of cellular functions, including cell survival, growth, motility, proliferation, and metabolism. As a result, manipulating PI3K-mediated alterations has become an attractive target for cancer therapy, and great efforts are underway to develop effective PI3K inhibitors. In this regard, BYL719, a pan-PI3K inhibitor, is being tested in clinical trials for esophageal squamous cell carcinoma (ESCC), metastatic head and neck squamous cell carcinoma, gastrointestinal stromal tumours, breast cancer, and ovarian cancer [10, 11]. However, it is difficult to conduct in-depth research because of the emergence of drug resistance.

Therefore, in this study, we explored the anticancer activity and potential mechanisms of casticin using nasopharyngeal carcinoma cells and investigated the molecular targeting of this chemical compound by using in silico prediction methods and cell function assays [12, 13]. We found that casticin could represent a novel and broadly applicable PI3K inhibitor that can be used for treatment against nasopharyngeal carcinoma cells and that casticin may provide potential clinical benefits for patients afflicted with nasopharyngeal carcinoma.

## Materials and methods

### Drugs

We purchased casticin from Chengdu Bio-purify Phyto-chemicals, Ltd. (Chengdu, China) and dissolved it in dimethyl sulfoxide (DMSO). The chemical inhibitor BYL719 was purchased from Selleckchem (Houston, TX, USA).

### Cell lines and cell culture

NPC cell lines 5-8F, 6-10B, CNE1, HNE1, S18, S26, HONE1, HONE1-EBV, SUNE1, and NP69 were obtained from the Cancer Research Institute of Southern Medical University (Guangzhou, China), and the CNE2 cell line was purchased from Keygen Biotech (Nanjing, China). The EBV-positive nasopharyngeal carcinoma cell line C666-1 was kindly provided by Professor Xin Li, Southern Medical University, and Professor G. S. W. Tsao, University of Hong Kong. All cell lines were confirmed to be mycoplasma free before the start of experiments. All nasopharyngeal carcinoma cell lines were cultured in RPMI-1640 medium (Invitrogen, USA) supplemented with 10% foetal calf serum (FCS; Gibco, Invitrogen), 100 U/mL penicillin, and 100 μg/mL streptomycin. NP69 cells were cultured in defined KSFM supplemented with epidermal growth factor (Invitrogen, Carlsbad, CA, USA) and 10% FCS. All cell lines were cultured in a humidified chamber with 5% CO_2_ at 37 °C.

### Cell viability assay

The effects of casticin on the growth of the 12 cell lines were determined using the Cell Counting Kit-8 (CCK-8) assay (Dojindo, Japan). Briefly, cells were plated in 96-well plates at 4 × 10^3^ cells/well in 100 μL and were treated with a gradient of casticin concentrations (0, 1, 2, 4, 8 µM). After incubation for 24, 48, or 72 h, 10 μL of the CCK-8 reagent was added into each well, and the plate was incubated for an additional 1 or 2 h. Next, a microplate reader (Bio-Rad, model 550) was used to detect absorbance at 490 nm in each well. We repeated the experiments three times. Cell viability was calculated using the following formula: cell viability = (OD of control − OD of treatment)/(OD of control − OD of blank) * 100%.

### Wound-healing assay

We seeded S18 and C666-1 cells in 6-well plates at 80% to 90% confluence. After a 24-h incubation period, cells were wounded by using a 0.2-mL pipette tip to scratch the subconfluent cell monolayer. Then, we removed the detached cells by washing with PBS just before casticin 16 µM was added to each well of the plate. Cells were maintained in serum-free medium and allowed to migrate for 24 h. An inverted microscope was used to capture cell migration images after 24 h. The speed of migration was determined by dividing the length of the gap and the wound areas in the resultant images.

### Colony formation assay

S18 and C666-1 cells were incubated in 6-well plates with either 0, 1, 2, and 4 µM casticin for 12 h with 1 µM casticin for 0, 6, 12, and 24 h. There after, 500 cells were seeded in each well of a 6-well plate and cultured without casticin for 8–10 days. Subsequently, the cells were fixed with methanol and stained with haematoxylin. After washing and drying, colonies with more than 50 cells were counted under a microscope (Leica, Wetzlar, Germany).

### Cell cycle analysis

S18 and C666-1 cells were treated with 0, 2, 4, and 8 µM casticin for 24 h. The cells were then collected, washed with phosphate-buffered saline (PBS), and fixed with 75% ethanol overnight. Subsequently, the cells were centrifuged (1500 rpm, 5 min) and incubated with 10 mg/mL RNase and 1 mg/mL propidium iodide (PI) at 37 °C for 30 min in the dark. Finally, we analysed cell cycle distribution by using flow cytometry (FACSCalibur; BD Biosciences, Bedford, MA, USA).

### Annexin V/PI staining assay

For the apoptosis test, S18 and C666-1 cells were treated with casticin at concentrations of 0, 1, 2, and 4 µM and of 0, 2, 4, and 8 µM for 48 h. Next, cells were collected and washed once with PBS. After centrifugation (1500 rpm, 5 min), cells were resuspended in 1× annexin V (AV) binding buffer and incubated with 5 µL of AV and PI at 37 °C for 30 min. Cell apoptosis was evaluated by using flow cytometry (FACSCalibur).

### Establishment of xenograft mouse model

Generation and characterization of the xenograft mouse model were conducted in accordance with the guidelines of the Ministry of Science and Technology of China and were approved by the Animal Ethics Committee of Southern Medical University. All efforts were made to minimize animal suffering during the experiments. Five-week-old nude mice were obtained from the Guangdong Experimental Animal Ccenter. To assess the tumour growth, 5 × 10^6^ S18 cells and 1 × 10^7^ C666-1 cells were injected subcutaneously into the left or right side of the back of each mouse (three mice per group). When tumours reached approximately 100 mm^3^, the mice were randomized to the control and casticin treatment groups. Casticin was diluted in 10% DMSO in normal saline (NS) and injected intraperitoneally at 40 mg/kg daily, while the control group received only NS vehicle. Tumour sizes were measured with a caliper and recorded every 3 days [7]. Tumour volume was calculated as follows: tumour volume = (length × width^2^) × 0.5. Body weight was recorded every 2–3 days during the treatment, and the tumour weight was measured at the end of the experiment.

### Tumour sphere formation assay

S18, CNE2, and C666-1 cells (2 × 10^4^ each) were cellected, counted and seeded into a cell culture flask with a novel 3D tumour sphere culture system [14]. The 3D tumour sphere culture system with NPC cells was cultured at 37 °C in a humid atmosphere with 5% CO_2_, the diameters of tumour spheroids were measured when the tumour sphere diameter was ≥ 40 µM [15] and then incubated with 20 µM casticin for 72 h, the original medium was discarded, the cells were cultured for approximately 1 week, and the tumour spheres that were ≥ 100 µm in diameter were counted under a bright field microscope.

### Western blotting

We used standard approaches and methods for Western blotting (WB) analyses. In brief, nasopharyngeal carcinoma cells were lysed on ice in Pierce RIPA buffer (Thermo Scientific) containing a Halt protease inhibitor cocktail (Sigma-Aldrich) and phosphatase inhibitors (Keygen Biotech). We measured protein concentration using a Beyotime protein assay protocol following the manufacturer’s instructions. Proteins were separated by using sodium dodecyl sulfate polyacrylamide gel electrophoresis and were then transferred to polyvinylidene difluoride membranes (0.45 µm; Millipore). We incubated membranes with primary antibodies that have demonstrated activity against the following targets: PI3K (4249 s, CST), phospho (p)-PI3K (11508; Enogene, China), mechanistic target of rapamycin (mTOR; 2927T, CST), p-mTOR (S2448) (ab109268, Abcam), p-mTOR (S2481) (ab137133, Abcam), 3-phosphoinositide-dependent protein kinase 1 protein kinase B (also known as AKT) (ab188099, Abcam), p-AKT (T308) (13038P; CST), p-AKT (S473) (ab81283, Abcam), p-S6 (S235/236) (4858T, CST), p-S6 (S240/244) (5364S, CST), S6 (2217S, CST), BCL-2 (ab182858; Abcam), BAX (ab32503, Abcam), CDK4 (ab108357, Abcam), CDK1 (ab133327, Abcam), cyclin B (ab32053, Abcam), p21 (ab109520, Abcam), CCND1 (ab134175, Abcam), Nanog (ab109250, Abcam), SOX2 (ab97959, Abcam), and OCT4 (ab109183, Abcam). The total S6, AKT, and mTOR antibodies were used on the same blots as the respective phosphorylated proteins after stripping (stripping buffer, CWBIO, China). All primary antibodies were diluted in Tris-buffered saline containing Tween 20 and 5% bovine serum albumin (BSA), and a peroxidase conjugate (Bioworld) was used as the secondary antibody. Anti-β-actin (20536-I, Proteintech) was used as a loading control. Immuno-reactive bands were visualized using an enhanced chemo-luminescence kit (Thermo Fisher Scientific), and images were captured using a ChemiDoc™ XRS molecular imager (Bio-Rad). Densitometry of band intensity was performed using ImageJ software.

### In silico target prediction and analysis of casticin binding by molecular docking

Chemical proteomics was used to uncover the target of casticin, while reverse docking and molecular dynamic simulation were performed to evaluate the binding site of casticin. Prediction of anticancer targets of casticin was carried out using ChemMapper [16] (http://59.78.96.61:8080/chemmapper/help.html), and the ROCS-Based Target Prediction database [17] (http://targetfishing.molcalx.com.cn/cancer.html) as shown in Tables 1 and 2. Binding analysis was performed by exploring PI3K protein structures available in the Protein Data Bank (PDB). Casticin (CID: 5315263) spatial data were retrieved from PubChem (http://www.ncbi.nlm.nih.gov/pccompound). Molecular docking and visualization of interactions were carried out using LigandScout [18] (verison4.1. bulid 2016-12-22), which was also used to calculate the binding energy. Ligands and water molecules were discarded, and hydrogen atoms were added to the proteins.

**Table 1:** The top 10 protein targets by reverse docking through 2D vs. 3D similarity with casticin

(Chemmapper) target name	Rank	Score	Uniprot
Proto-oncogene serine/threonine-protein kinase Pim-1	1	1	P11309
Estrogen receptor beta	2	0.894	Q92731
Phosphatidylinositol-4,5-bisphosphate 3-kinase	3	0.639	P48736
Cell division protein kinase 2	4	0.408	P24941
Estrogen receptor	5	0.407	Q61026
HpFabZ	6	0.351	NONE
QacR (E58Q)	7	0.27	P0A0N3
Dihydroflavonol 4-reductase	8	0.236	P51110
Reticuline oxidase; berberine bridge-forming enzyme	9	0.226	P30986
Reticuline oxidase	10	0.217	P30986

**Table 2:** The top 10 protein targets of reverse docking through molecular and pharmacodynamic shape with casticin

(ROCS BASED) Protein name	Rank	Tanimoto Combo	ShapeTanimoto
Phosphatidylinositol 4,5-bisphosphate 3-kinase catalytic subunit gamma isoform	1	1.535	0.894
Phosphatidylinositol 4,5-bisphosphate 3-kinase catalytic subunit alpha isoform	2	1.137	0.752
Mitogen-activated protein kinase 14	3	1.136	0.843
Serine/threonine-protein kinase GSK3B	4	1.113	0.839
Phosphatidylinositol 3-kinase catalytic subunit type 3	5	1.097	0.766
Acetylcholinesterase	6	1.092	0.764
cAMP-specific 3′,5′-cyclic phosphodiesterase 4D	7	1.012	0.74
Cyclin-dependent kinase 5	8	0.968	0.746
Amine oxidase [flavin-containing] B	9	0.965	0.762
High affinity cGMP-specific 3′,5′-cyclic phosphodiesterase 9A	10	0.921	0.685

### KINOMEscan assay and binding affinity test

Selectivity profiling of casticin against a selected panel of 468 kinases distributed throughout the kinome was performed by DiscoverRx (http://www.discoverx.com/home) using the KINOMEscan screening platform at a test concentration of 10 μM. Since KINOMEscan is a binding assay and may not reflect the inhibitory activity of a compound, the binding constant (*K*_d_) of casticin was calculated from a standard dose–response curve using the Hill equation: Response = Background + (Signal − Background)/[1 + (*K*_d_^Hill Slope^/Dose^Hill Slope^)]. We then tested casticin in the SelectScreen assay (Invitrogen, USA) to calculate the IC50 of casticin that directly targeted the active site of ATP in PI3K kinase.

### Statistical analysis

Statistical analysis was performed using SPSS 20.0 statistical software. All experiments were repeated at least three times. The CCK-8 data are shown as the mean ± SEM, and the other experimental data in the figures and text are shown as the mean ± standard deviation. The results from the wound healing, cell cloning, Transwell, tumour spheroid formation, and flow cytometry assays were analysed using the two independent samples t-test; Western blotting and CCK-8 data were compared using one-way analysis of variance (ANOVA), followed by multiple comparisons tests using the LSD method if the variance was homogenous; if the variance was not homogenous, the Welch method was used to approximate variance, and multiple comparisons were analysed using the Dunnett T3 method. *p* < 0.05 was considered statistically significant.

## Results

### Casticin inhibits the viability and proliferation of nasopharyngeal carcinoma cells

Eleven NPC cell lines and the normal nasopharyngeal mucosal epithelial cell line NP69 were used to characterize the effects of casticin on cell growth (Fig. 1b; Additional file 1: Fig. S1a). Cells were treated with a gradient of casticin concentrations at different time points (24, 48, and 72 h), and cell viability was assessed by CCK-8 assay. Casticin decreased the viability of eleven NPC cell lines in a concentration-dependent manner, while NP69 cells were found to be resistant (Fig. 1b). We used S18, which is one of low-differentiation nasopharyngeal carcinoma cell lines, and C666-1, which is EBV^+^ as models of the cell growth inhibition induced by casticin in subsequent experiments. A wound-healing assay was performed to evaluate the effect of casticin on NPC cell migration, and the results revealed that gap-closing speeds were significantly decreased in S18 and C666-1 cells treated with casticin compared to cells that were not treated with casticin (Fig. 1c). In the Transwell migration assay, the number of migrating cells was significantly reduced in the casticin-treated groups in a concentration-dependent manner (Fig. 1d). These results indicated that casticin could inhibit NPC cell migration. The colony formation assay is widely used for evaluating cancer cell survival and proliferation [19]. A significant reduction in the clonogenic activity of S18 cells were exposed to 0, 1, 2, and 4 µM casticin for 12 h (Fig. 1e). Moreover, the clonogenic ability was diminished as early as 6 h after the treatment with 1 µM casticin compared with that derived from the control group (Fig. 1f). These results demonstrated that casticin could inhibit the proliferation of NPC cells in a concentration- and time-dependent manner.

### Casticin inhibits tumour growth in a xenograft mouse model

To evaluate the anti-tumour activity of casticin in vivo, we injected the xenograft mice with casticin intraperitoneally at a dose of 40 mg/kg daily for 12 consecutive days. In the S18 model, we found that the tumours in the mice treated with casticin were smaller than those in the control group in samples taken 18 days after implantation (Fig. 1g). Tumour volume was periodically measured for each mouse, the tumour growth rate in the casticin treatment group was lower than NS group (Fig. 1h), tumours were excised from the nude mice and weighed, tumour in the treatment group were lighter than NS group (Fig. 1i).

Next, rations of liver and kidney weight to body weight in three different groups of the S18 were calculated, not statistically significant between groups (Additional file 2: Fig. S2c); we checked the histopathology of the liver and kidney by using haematoxylin and eosin stainning and found that the renal tubular morphology of the kidney was normal in both groups and that the liver cells had uniform cytoplasms (Additional Fig. 2d). These results indicate that casticin treatment can effectively inhibit tumour growth in mice without any obvious subsequent damage to the liver or kidney. The results from the C666-1 in vivo model are shown in Additional file 2: Fig. S2e–g, indicating findings similar to those with the S18 model.

**Fig. 2:**
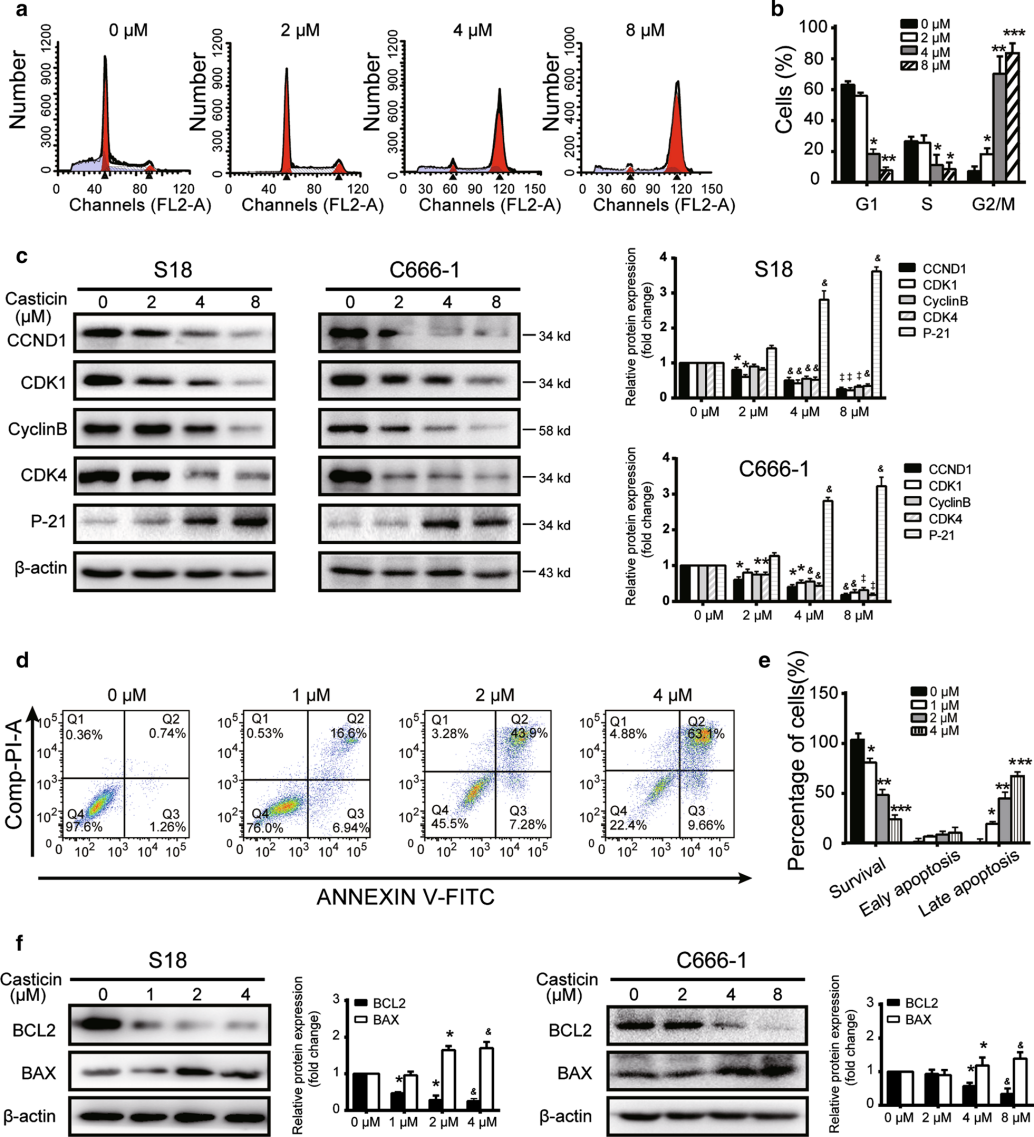
Casticin induces G2/M cell cycle arrest and apoptosis in NPC cells. **a**, **b** S18 cells were treated with casticin at concentrations of 0, 2, 4 or 8 µM for 24 h. Cell cycle distribution was analysed by flow cytometry. All data are presented as the mean ± standard deviation. *p < 0.05 versus 0 µM, **p < 0.01 versus 2 µM, and ***p < 0.001 versus 4 µM. **c** Levels of expression of cyclinD1, CDK1, cyclin B, CDK4, p21 were determined using Western blotting analysis in S18 and C666-1 cells after application of a gradient of casticin concentrations for 24 h. All data are presented as the mean ± standard deviations. *p < 0.05 versus 0 µM, ^&^*p* < 0.5 versus 2 µM, and ^‡^*p* < 0.05 versus 4 µM. **d**, **e** Cell apoptosis was determined by flow cytometry after treatment with casticin at concentrations of 0, 1, 2, and 4 µM for 48 h. All data are presented as the mean ± standard deviation. *p < 0.05 versus 0 µM, **p < 0.01 versus 1 µM, and ***p < 0.001 versus 2 µM. **f** Levels of expression of apoptosis related proteins BCL2 and BAX in S18 cells with a gradient of casticin concentrations (0, 1, 2, 4 µM), and C666-1 with a gradient of casticn concentrations (0, 4, 8 or 16 µM) analyzed by Western blotting. All data are presented as the mean ± standard deviation, for S18 cells *p < 0.05 versus 0 µM, ^&^*p* < 0.5 versus 1 µM; for C666-1 cells *p < 0.05 versus 0 µM, ^&^*p* < 0.5 versus 4 µM. All the assays were performed in triplicate

### Casticin induces apoptosis and G2/M phase arrest in S18 and C666-1 cells

It has previously been reported that casticin can induce G2/M phase arrest in multiple types of cancer cells [20, 21]. We found that casticin had a similar effect on nasopharyngeal carcinoma cells, wherein treatment resulted in the accumulation of cells in the G2/M phase of the cell cycle in a dose-dependent manner (S18 cells as an example: Fig. 2a, b) and found a similar phenomenon in C666-1 cells (Additional Fig. 2h, i). The percentage of S18 and C666-1 cells in the G2/M phase dramatically increased from 11.64 ± 4.72 and 7.03 ± 2.24%, respectively, in the untreated control to 62.90 ± 1.97 and 62.54 ± 4.91%, respectively, after treatment with 8 µM casticin for 24 h; that the percentage in the treated group was still significantly higher than that in the untreated group (*p* < 0.05). To investigate the mechanism of casticin-induced cell cycle arrest, we examined the expression levels of cell cycle-related proteins, cyclin D1-dependent CDK4 and CDK1 (Fig. 2c); changes in expression were consistent with an indication of G2/M cell cycle arrest (Fig. 2a, b, Additional file 2: Fig. S2h, i). Previous studies have also indicated that p21 can induce cell cycle arrest at the G2/M phase and that the PI3K/AKT pathway plays an important role in regulating cell cycle progression and cell proliferation via p21. Accordingly, we also found an increased level of p21 expression after casticin treatment (Fig. 2c).

To elucidate whether apoptosis was also involved in the nasopharyngeal carcinoma cell growth induced by casticin, we examined the degree of apoptosis in casticin-treated S18 and C666-1 cells by using the AV/PI assay. Casticin-treated S18 cells had significantly increased early and late apoptosis even at concentrations of only 1 μM, and additionally, the apoptotic frequency was as high as approximately 75% at 4 μM (Fig. 2d, e). We found a similar trend for C666-1 cells (Additional file 2: Fig. S2j, k). We also observed that B-cell lymphoma 2 (BCL-2)-associated X protein BAX was upregulated, while BCL-2 was downregulated in the casticin-treated cells, and the BCL-2/BAX ratio was significantly decreased in a dose-dependent manner (Fig. 2f).

### Casticin inhibits nasopharyngeal carcinoma stem cell characteristics

**Fig. 3:**
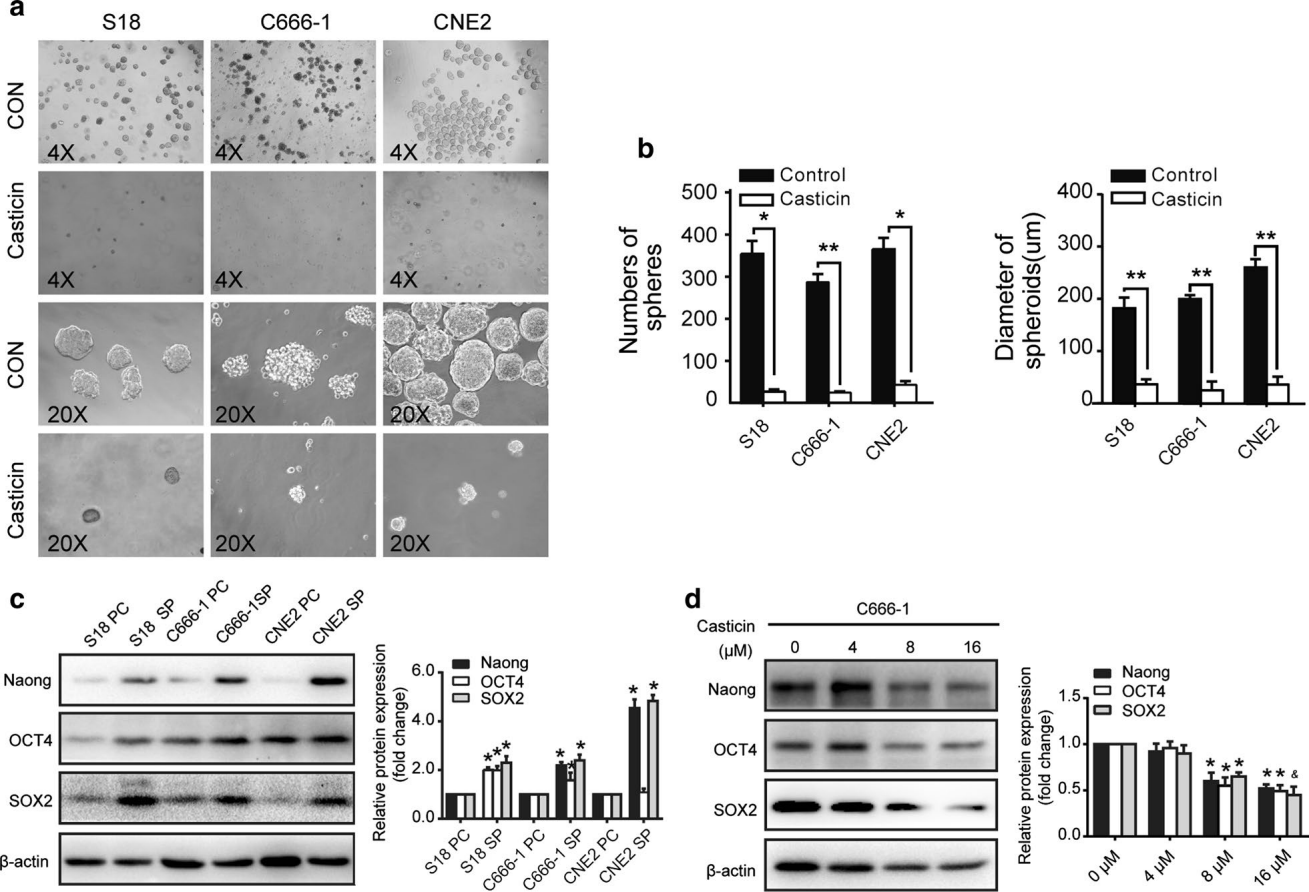
Casticin inhibits the formation of tumour spheres derived from NPC cells. **a** S18, CNE2, C666-1 cells were induced to generate tumour spheres and were treated with 20 µM casticin for 72 h. **b** The number and diameter of spheres of different groups are shown in the right panels. All data are presented as the mean ± standard deviation. *p < 0.05 versus control, and **p < 0.01 versus control. **c** Western blotting showing the expression levels of stem cell marker proteins, such as NANOG, OCT4, and SOX2 which were examined in tumour spheres (SP) and compared with their parental cells (PC). All data are presented as the mean ± standard deviation. *p < 0.05 versus PC. **d** The levels of expression of NANOG, SOX2, and OCT4 in C666-1 cells were examined after treatment with a gradient of casticin concentrations (0, 4, 8 or 16 µM) for 24 h. All data are presented as the mean ± standard deviation. *p < 0.05 versus 0 µM, and ^&^*p* < 0.05 versus 8 µM

To characterize the effect of casticin on nasopharyngeal carcinoma stem cells, we produced nasopharyngeal carcinoma tumour spheres using a new three-dimensional (3D) tumour sphere culture system, which was successfully established in our laboratory [14]. At a density of 20,000 cells per culture flask, we found that increased numbers of spheres were formed in the S18, CNE2, and C666-1 cell cultures, we examined whether casticin could affect the self-renewal ability of nasopharyngeal carcinoma cell lines (Fig. 3a). After treatment with 20 µM casticin for 72 h, a significant reduction in tumour growth was observed in the casticin-treated tumour spheres compared with that in the untreated spheres (Fig. 3b). Although the size of tumour spheres varies depending on the founder clone, stem cells are believed to give rise to larger spheres (typically, spheres from 40 to 150 μm in diameter qualify as tumour spheres), while progenitors give rise to smaller spheres. We evaluated the level of expression of stemness-associated proteins using WB to verify whether the new culture system was able to enrich the cancer stem cell characteristics of nasopharyngeal carcinoma cells. The results revealed that the protein expression levels of NANOG, OCT4, and SOX2 were significantly higher in tumour spheres than in their parental cells (Fig. 3c). Subsequently, we examined the protein expression of stem cell markers, including Nanog, sox2, and Oct4, in nasopharyngeal carcinoma cell lines. As shown in Fig. 3d, casticin-treated nasopharyngeal carcinoma cells displayed reduced expression of these three tumour stemness makers in a dose-dependent manner. Based on these criteria, our results showed that casticin can inhibit the tumour sphere formation in NPC cell lines.

### Identification of casticin target proteins

To predict protein targets of casticin, a combined strategy was used. We conducted in silico screening by using different reverse docking approaches [17, 22]. We screened casticin against the ChemMapper database to find candidate targets based on 2D/3D structural similarity (Table 1).

Additionally, the ROCS-Based Target Prediction database was used, which allows the prediction of potential targets based on the ligand shape and electrostatics (Table 2).

**Fig. 4:**
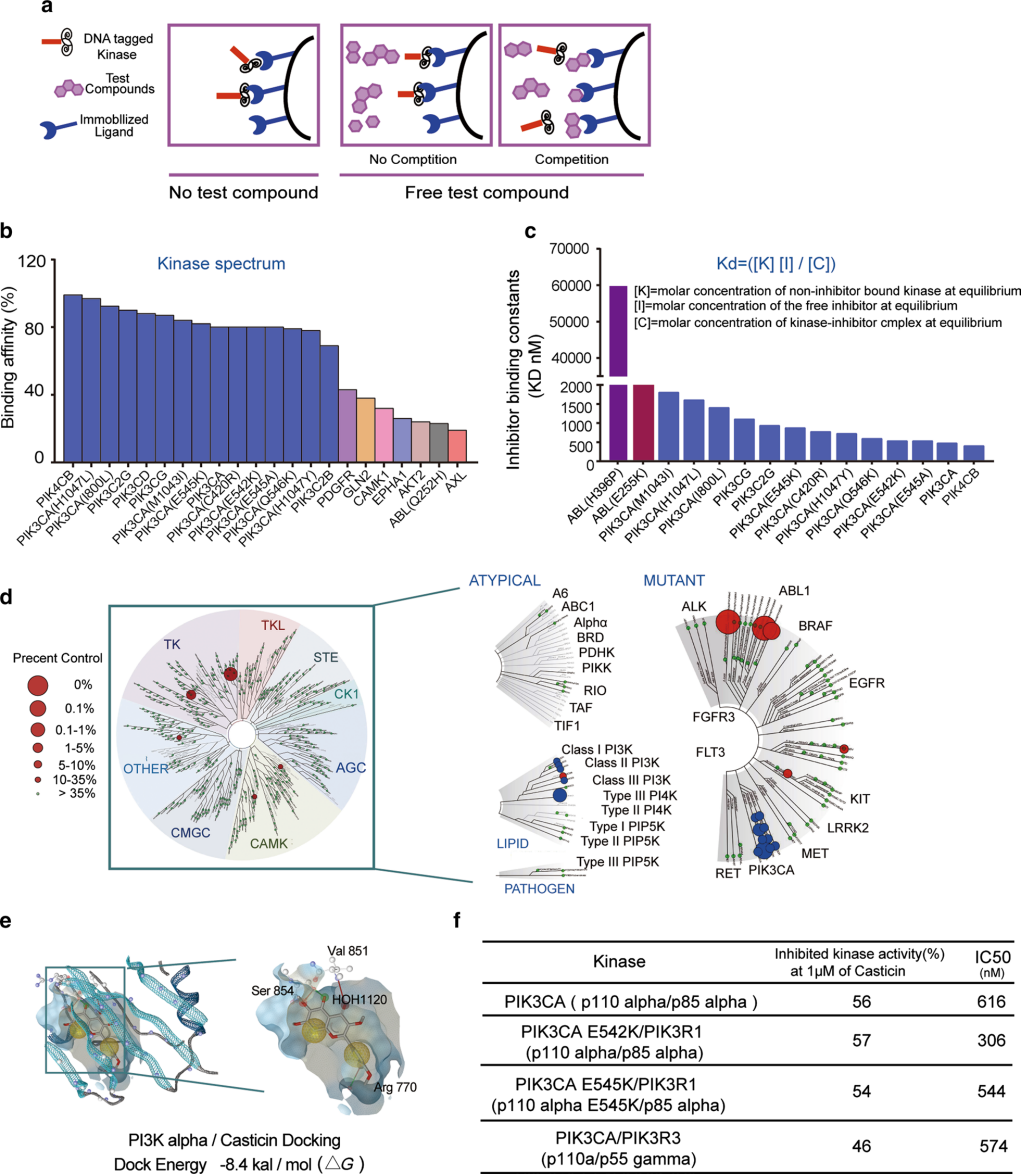
Casticin is a selective inhibitor of PI3K. **a** Schematic illustration of the KINOMEscan platform and high-throughput kinase profiling technology. The phage-tagged kinase is shown in red, ‘free’ test compound in purple and immobilized ‘bait’ ligand in blue. **b** Kinome-wide selectivity profiling of casticin analysed through KINOMEscan. **c** Binding constants (K_d_s) were calculated with a standard dose–response curve using the Hill equation shown in this panel. **d** Kinome-wide selectivity profiling of casticin was analysed through KINOMEscan. The data shown are representative of one of at least three independent experiments. **e** Computational docking of casticin into each active pocket of 4I2Y (PI3K110α) and docking energy obtained from casticin binding the crystal structure of PI3K110α. **f** Invitrogen SelectScreen™ biochemical kinase activity assay with the indications for the best potential targets of casticin

To validate the predicted targets, we tested casticin against a panel of 468 wild-type and mutant kinases included in the DiscoveRx KINOME scan profiling platform (Fig. 4a), which covers more than 80% of the human catalytic protein kinome (Fig. 4a). At a concentration of 10 μM, casticin showed high and selective targeting with an S score (1) of 0.01 (Fig. 4b). The binding *K*_d_ values, determined with microscale thermophoresis technology, indicated strong binding of casticin to the PI3K family (Fig. 4c). The strong binding (with the percent control number of less than 1) of casticin to enzymes of the PI3K family, including wild-type and mutant kinases (Fig. 4d), indicated casticin as a highly selective inhibitor of PI3Ks. Casticin, however, did not demonstrate strong binding to other kinases in the KINOME scan assay. To understand the interaction of casticin with binding site residues of PI3Kα [23], casticin was docked to the protein crystal structure of PI3K (Fig. 4e). The crystal structures of PI3K (3HHM) were obtained from the PDB (http://www.rcsb.org/pdb/home/home.do) and the flexible docking of casticin with the PI3K kinase domain was performed with the standard precision mode [12, 17]. The results showed that casticin formed hydrogen bonds with PI3K at Val854 and Agr770, and a hydrophobic bond is formed at Val851. The docking score of casticin against the kinase domain of PI3K, expressed as free energy (ΔG; kcal/mol), was − 8.4 (Fig. 4e). We used the Invitrogen SelectScreen™ Biochemical Kinase Activity Assay to evaluate the inhibitory activity of casticin. Among the currently available purified proteins, casticin inhibited PI3K (p110α/p85α) with an IC_50_ value of 616 nm (Fig. 4f). These results indicated that casticin is a novel inhibitor of both wild-type and mutant PI3K.

### Screening of BYL719-insensitive cell lines of NPC

**Fig. 5:**
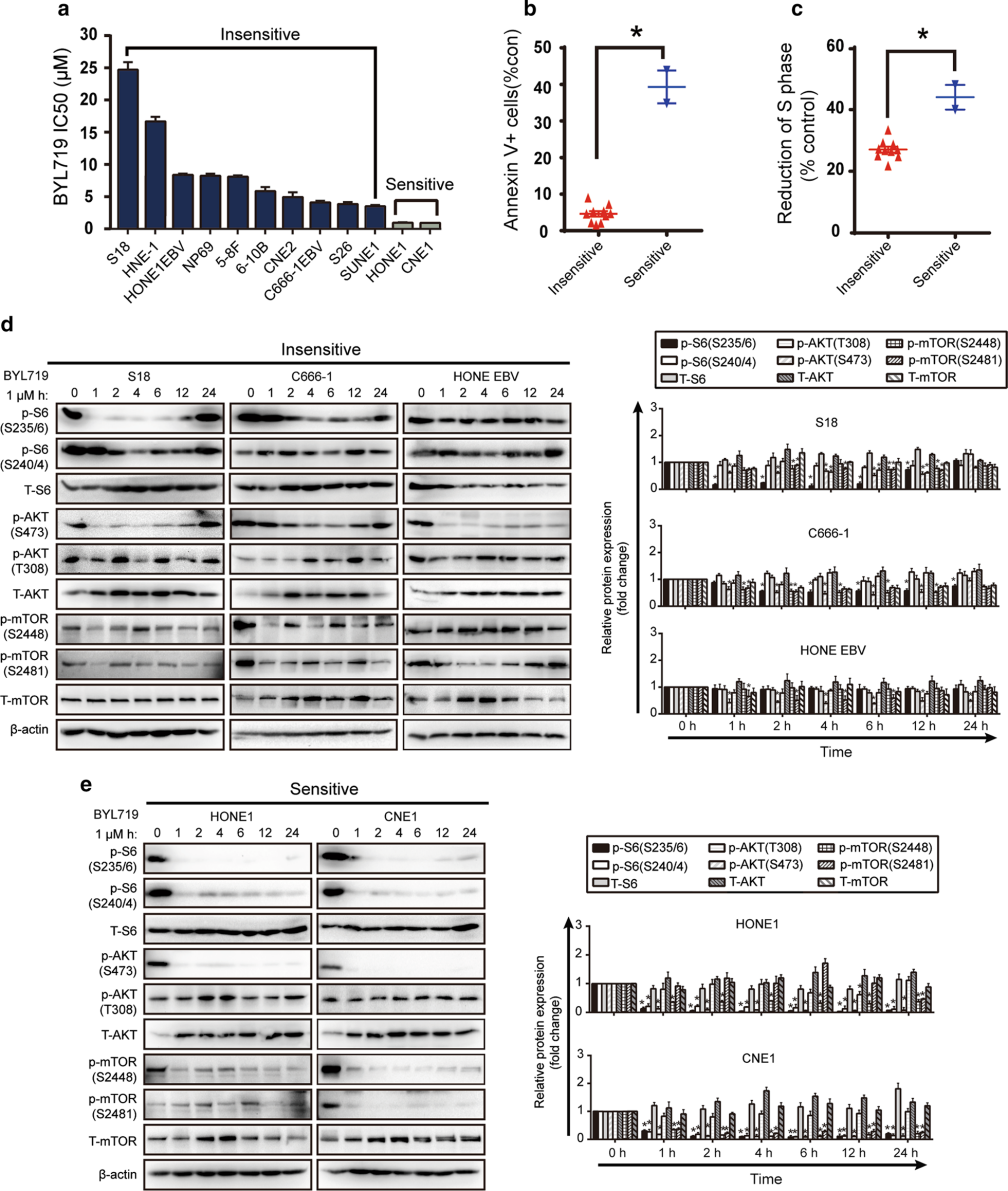
Casticin inhibits the PI3K/mTOR/AKT pathway in BYL719-insensitive cells. **a** Measurement of the IC50 of BYL719 in 12 NPC cell lines after 72 h of treatment by examining its effects on viability. Values are shown as the mean ± standard error of the mean (SEM). Data points represent combined values across triplicate samples. **b** Apoptosis (annexin V) in BYL719-sensitive and BYL719-insensitive NPC cells after 48 h of treatment with 1 μM BYL719. Each dot represents the mean of three independent experiments, each performed in duplicate per listed cell line. *p < 0.05 versus insensitive. **c** After treatment with 1 μM BYL719 for 48 h, sensitive cell lines had, on average, a larger proportion of cells in cell cycle arrest (S-phase arrest) than did insensitive lines. Each dot represents the mean of three independent experiments, each performed in duplicate per listed cell line. *p < 0.05 versus insensitive. **d**, **e** Western blotting results showing PI3K pathway signalling in BYL719-insensitive resistant and BYL719-sensitive NPC cell lines. Protein lysates from cells treated with 1 μM BYL719 were extracted at different time points and analysed by Western blotting with the indicated antibodies. Densitometric analysis of Western blot bands is shown in the right panel. All data are presented as the mean ± SD, *p < 0.05 versus 0 h

To identify BYL719-insensitive NPC cell lines, the response of 12 NPC cell lines to BYL719 was screened by viability assay (Fig. 5a). At 1 μM, BYL719 blocked p110α without affecting the other p110 subunits, thus, we used an IC_50_ value of 1 μM as a cut-off to classify NPC cell lines as BYL719-sensitive or -insensitive [24]. Only two NPC cell lines, HONE1 and CNE1, were sensitive to 1 µM BYL719 after 72 h (Fig. 5a). Both sensitive cell lines increased S-phase arrest and apoptosis rates (as measured by AV staining) compared with the those in the BYL719-insensitive cell lines (Fig. 5b, c). We studied PI3K signalling in three resistant (S18, C666-1 and HONE-EBV+) (Fig. 5d) and two sensitive cell lines (HONE1 and CNE1) after treatment with a gradient of BYL719 concentrations (Fig. 5e). The results revealed that the phosphorylation of S6, AKT and mTOR was significantly reduced in the sensitive cells compared to that in the resistant cells, which suggested that BYL719 can potentially be used in NPC treatment. The mild reduction of PI3K phosphorylation observed in the resistant cells explained the higher viability of these cells after BYL719 treatment.

### Casticin selectively inhibits the PI3K/AKT/mTOR signalling pathway

**Fig. 6:**
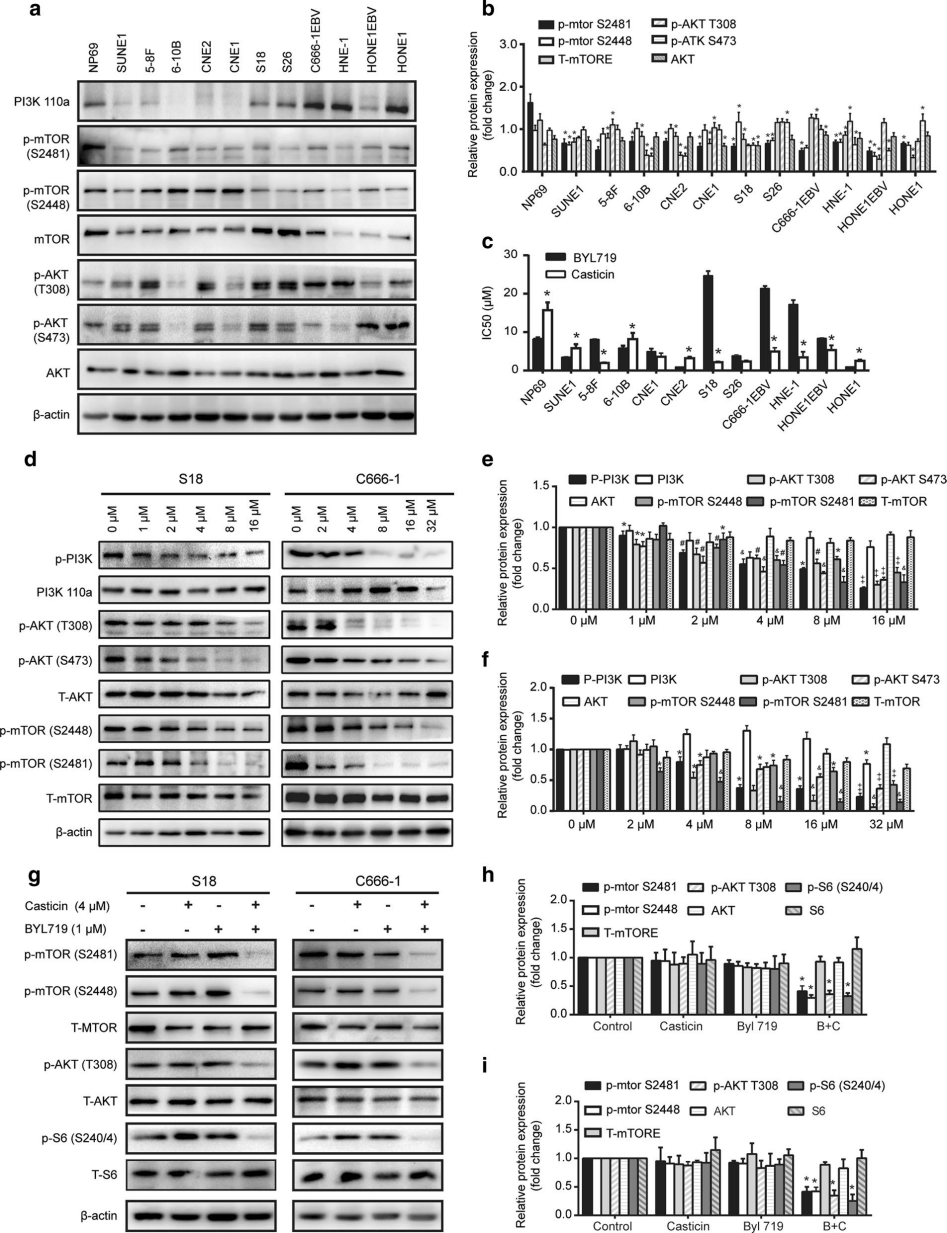
Casticin inhibits the PI3K/mTOR/AKT pathway in BYL719-resistant cells. **a**, **b** Western blot analysis of the basal expression of PI3K110α, p-mTOR, mTOR, p-AKT, and AKT in a panel of twelve NPC cell lines. All data are presented as the mean ± standard deviation. *p < 0.05 versus NP69. **c** IC50 values for casticin- and BYL719-induced effects on the viability of 12 NPC cell lines after 24 h of treatment. All data are presented as the mean ± standard deviation, *p < 0.05 versus BYL719. **d**–**f** Western blot analysis of PI3K/AKT signalling in cells treated with different concentrations of casticin for 24 h. **d** Western blotting analysis of PI3K/AKT signalling in cells treated with different concentrations of casticin for 24 h. **e**, **f** Densitometric analysis of Western blot bands of left panel. **e** for S18 cells, *p < 0.05 versus 0 µM, ^**#**^*p* < 0.5 versus 1 µM, ^&^*p* < 0.5 versus 2 µM, ^‡^*p* < 0.05 versus 4 µM; **f** for C666-1 *p < 0.05 versus 0 µM, ^&^*p* < 0.5 versus 2 µM, ^‡^*p* < 0.05 versus 4 µM. **g** BYL719-insensitive cells. S18 and C666-1 were treated with 4 µM casticin alone or combined with 1 µM BYL719 for 12 h. Whole cell extracts were prepared and subjected to WB analysis using antibodies against p-AKT (T 308), p-mTOR (S2448), p-mTOR(S2481) and p-S6(S240/4). All data are presented as the mean ± standard deviations. *p < 0.05 versus control

The PI3K/AKT signalling pathway plays an important role in cancer proliferation and metastasis and is frequently activated in cancer tissues, including nasopharyngeal carcinoma [25]. First, we evaluated the expression and phosphorylation of the PI3K subunit 110α, encoded by PIK3CA, and its downstream effectors AKT and mTOR in all 12 NPC cell lines (Fig. 6a, b). Five cell lines, including S18, S26, C666-1 EBV^+^, HNE-1 and HONE-1, expressed high levels of the PI3K subunit. We compared the effect of casticin and BYL719 on cell growth in 12 NPC cell lines and found that the IC50 ratio of casticin was lower than that of BYL719 (Fig. 6c). Compared to BYL719, casticin was less effective in NP69 cells. Next, we observed a significant reduction in phosphorylation of AKT at T308 and S473 sites, as well as phosphorylation of mTOR at Ser2481 and Ser2448, in both S18 and C666-1 after 24 h of application of increasing casticin concentrations, and the effects occurred in a dose-dependent manner (Fig. 6d–f). However, there was no difference in total PI3K and AKT expression, indicating that the reduced phosphorylation was not due to a reduction in total protein expression (Fig. 6d–f).

### Casticin and BYL719 efficiently inhibit the PI3K/Akt/mTOR signalling pathway

We observed persistent mTORC1 signalling in BYL719-insensitive cells, which was consistent with the report that the activation status of mTORC1 is a determinant of drug sensitivity of PIK3CA mutant tumours. Then, we checked PI3K/AKT pathway signalling in S18 and C666-1 cells treated with BYL719 (1 µM) or casticin (4 µM) alone or combined for 12 h. The results showed that treatment with BYL719 alone did not reduce the phosphorylation of ATK (Ser473 and Thr308) or mTORC1. In contrast, the combination of the casticin with BYL719 decreased the phosphorylation of S6 at the Ser240/4 and Ser235/6 positions (Fig. 6g–i).

## Discussion

In this study, we explored the mechanism behind the antitumour activity of casticin and found that this compound is a selective broad-spectrum inhibitor against PI3K and its multiple mutants. Activation of the PI3K/AKT/mTOR pathway promotes cancer progression and resistance to endocrine therapy [26]. The *PIK3CA* gene is one of the nine commonly mutated genes in NPC, and it is activated by hot-spot mutations (e.g. those encoding His1047Arg, Glu545Lys, and Glu542Lys alterations) and amplified in 40–70% of NPC tissues [27]. Our study provides a pharmacological basis for the antitumour effects of casticin as a new therapy for nasopharyngeal carcinoma [28]. PIK3CA gain-of-function mutations are one of the common genetic aberrations in human malignancies, which makes PI3K an attractive target for cancer therapy. Despite the great promise of targeted therapy, treatment failure because of drug resistance remains an obstacle.

In recent years, increasing evidence has indicated the beneficial effects of selective PI3K inhibitors on NPC, suggesting that such inhibitors may offer novel therapeutic options for the treatment of the disease. Here, we demonstrated that the potent antitumour effect of casticin on NPC was mediated through the PI3K family, especially the PI3K110α subunit. Mechanistic studies revealed that casticin is a selective inhibitor against PI3K and its multiple mutants. Our results also indicated that casticin can serve as a candidate for the treatment of cancer patients who are resistant to PI3K inhibitor, such as BYL719. Importantly, this study provides a pharmacological basis for the antitumour effects of casticin in NPC. Casticin blocks the feedback activation of AKT caused by mTOR inhibition and directly blocks downstream PI3K multi-channel crosstalk, thereby preventing compensatory effects between different signalling pathways. Our results indicate that casticin as a selective pan-PI3K inhibitor, has a promising clinical application prospects. We also found that casticin was less cytotoxic to the immortal nasopharyngeal epithelial cell line NP69 and showed no significant hepatotoxicity in vivo. These properties make it an ideal candidate for cancer therapy.

Casticin is specific for and highly cytotoxic to the tumour spheres of nasopharyngeal carcinoma cells and represses the expression of stemness-related proteins, suggesting that casticin can inhibit the growth of nasopharyngeal carcinoma stem cells. Tumour stem cells (cancer stem cells, CSCs) can resist traditional cytotoxic chemotherapy and radiotherapy, which can promote the formation and infinite growth of tumour tissue. CSCs are considered to play an important role in tumour recurrence, metastasis and treatment tolerance. Therefore, CSCs that develop radiotherapy resistance are often noted as the main cause of recurrence and metastasis of NPC. Selective interventions targeting CSCs may be a new treatment option for NPC. The Sox2 gene is an important member of the Sox family and is located on chromosome 3q26.3•q27. It plays an important role in the transformation of pluripotent stem cells [28]. Nanog is another important stem cell transcription factor that together with Sox2, plays an important role in maintaining the multipotential differentiation potential of human embryonic stem cells and in determining the stage of cell differentiation during early embryonic development. Oct4 and Sox2, as key genes in ESC, do not act independently on the regulation of related pluripotency factors but form Oct4-Sox2 heterodimeric complexes. There is a bistable switch composed of Oct4-Sox2-Nanog that can be activated or inactived as the external environment changes and different signals are accordingly received [29]. Oct4, Sox2 and Nanog are essential transcription factors that help to maintain the ability of embryonic and adult stem cells to undergo self-renewal and multidirectional differentiation. In this study, we found that casticin was highly and specifically cytotoxic to the tumour spheres of NPC cells and suppressed the expression of stemness-related proteins SOX2, NANOG, and OCT-4, suggesting that casticin was able to inhibit NPC stem cells.

In summary, our findings show that casticin not only inhibits the stemness of NPC but also selectively inhibits PI3K and significantly suppressesNPC cell functions; we also showed that casticin in combination with BYL719 effectively reduced the phosphorylation of PI3K/AKT/mTOR proteins. This study is intriguing, as combinatorial antineoplastic effects of different flavonoids have been previously reported with various anticancer agents commonly used in the clinic. Overall, our data suggest that casticin can potentially be employed in combination therapy against NPC; however, further validation in preclinical studies is required.

## Conclusion

Casticin is a new selective PI3K inhibitor with targeted therapeutic potential for the treatment of NPC.

## Supplementary information

**Additional file 1: Fig. S1**. Casticin inhibits the viability, migration and invasion of NPC cells. **a** Ten NPC cell lines were treated with various concentrations of casticin for 24, 48 or 72 h. Cell viability was assessed using the CCK-8 assay. All the data are presented as the mean ± SEM, *p < 0.05 versus 0 µM; ^**#**^*p* < 0.05 versus 2 µM; ^&^*p* < 0.05 versus 4 µM; ^‡^*p* < 0.05 versus 8 µM. **b** IC50 values of casticin in 12 cell lines for 24, 48 or 72 h. **c** Wound-healing assay of C666-1 cells before and after casticin treatment. White dashed lines indicate the wound edge. The residual gap between the migrating cells from the opposite edges of the wound is represented as a percentage of the initial scratch area. Corresponding graphs show the mean width of the injury lines of three experiments (right). All data are presented as the mean ± standard deviation. *p < 0.01 versus DMSO. **d** Casticin-induced inhibition of C666-1 migration in the Transwell assay. Corresponding graphs (panel on right) show the mean numbers of cells per high-powerfield (HPF) from five independent areas. All data are presented as the mean ± standard deviation, and the representative experiment shown was repeated three times. *p < 0.05 versus 0 µM, **p < 0.01 versus 4 µM, and ***p < 0.001 versus 8 µM.

**Additional file 2: Fig. S2** Casticin inhibits the proliferation of NPC cells in vitro and in vivo and induces G2/M arrest and apoptosis in the NPC cell line C666-1. **a**, **b** Casticin suppressed colony formation of C666-1 cells. Cells were exposed to casticin 0, 1, 2, and 4 µM casticin for 12 h or were treated with 1 µM casticin for different time points (0, 6, 12, and 24 h), and were allowed to form colonies for approximately 10 days. Corresponding graphs show the mean number of colonies formed by different groups for three experiments (right). Right panel of a): *p < 0.05 versus 0 µM, **p < 0.01 versus 2 µM, and ***p < 0.001 versus 4 µM. Right panel of **b**: *p < 0.05 versus 6 h, **p < 0.01 versus 12 h, and ***p < 0.001 versus 24 h; **c** Ratios of liver and kidney weight to body weight in three different groups of the S18 nude mouse model; **d** HE staining of liver and kidney in the experimental group and saline group in regards to the tumorigenicity of the S18 mouse model. **e** Casticin inhibits tumour growth in vivo. Different concentrations (10% DMSO + 90% physiological saline, 40 mg/kg) of casticin were injected into nude mice once per day after they were inoculated with C666-1 cells. Images of 3 representative mice from each group are presented to show the sizes of the resulting tumours. **f** Tumour volume was periodically measured for each mouse and tumour growth curves were plotted. Data were used in a parametric generalized linear model with random effects (top of the panel). Tumours were excised from the animals and weighed (bottom of the panel). **g** HE staining of liver and kidney in the experimental and saline groups campared with the untreated group in the C666-1 nude mouse model. All data are presented as the mean ± standard deviation, and each experiment was repeated three times. *p < 0.05 versus NS. **h**, **i** C666-1 cells were treated with casticin at 0, 2, 4 or 8 µM for 24 h. The cell cycle distribution was analysed using flow cytometry; **j**, **k** Cell apoptosis was determined by flow cytometry after treatment with casticin at concentrations of 0, 2, 4, 8 µM for 48 h. The data shown are representative experiment from at least three independent experiments. All the assays were performed in triplicate. All data are presented as the mean ± SD. Significant differences compared with the control are indicated by *p < 0.05 versus 0 µM, **p < 0.05 versus 2 µM, and ***p < 0.05 versus 4 µM.

## Data Availability

Not applicable.

## Abbreviations

PI3K: phosphatidylinositol-3-hydroxykinase
AKT: protein kinase B
mTOR: rapamycin target protein
FBS: fetal bovine serum
NS: normal saline
