# Correction to: Casticin inhibits nasopharyngeal carcinoma growth by targeting phosphoinositide 3-kinase

**DOI:** 10.1186/s12935-021-01844-9

**Published:** 2021-02-26

**Authors:** Jingxian Liu, Jinghong Yang, Yuhe Hou, Zhenwei Zhu, Jie He, Hao Zhao, Xidong Ye, Dengke Li, Zhaohui Wu, Zhongxi Huang, Bingtao Hao, Kaitai Yao

**Affiliations:** 1 Guangdong Provincial Key Laboratory of Tumor Immunotherapy, Cancer Research Institute, School of Basic Medical Sciences, Southern Medical University, Guangzhou, 510515 Guangdong People’s Republic of China; 2 Shenzhen Hospital, Southern Medical University, Shenzhen, 518000 Guangdong People’s Republic of China; 3 Shunde Hospital, Southern Medical University, Shunde, 528300 Guangdong People’s Republic of China

## Correction to: Cancer Cell Int (2019) 19:348 10.1186/s12935-019-1069-6

Following publication of the original article [1], we were notified that the control group in the left panel of Figure 1c was incorrect, the labelling of the control group in Figure 1e was inconsistent.

Corrected Fig. 1 can be found below:

**Fig. 1:**
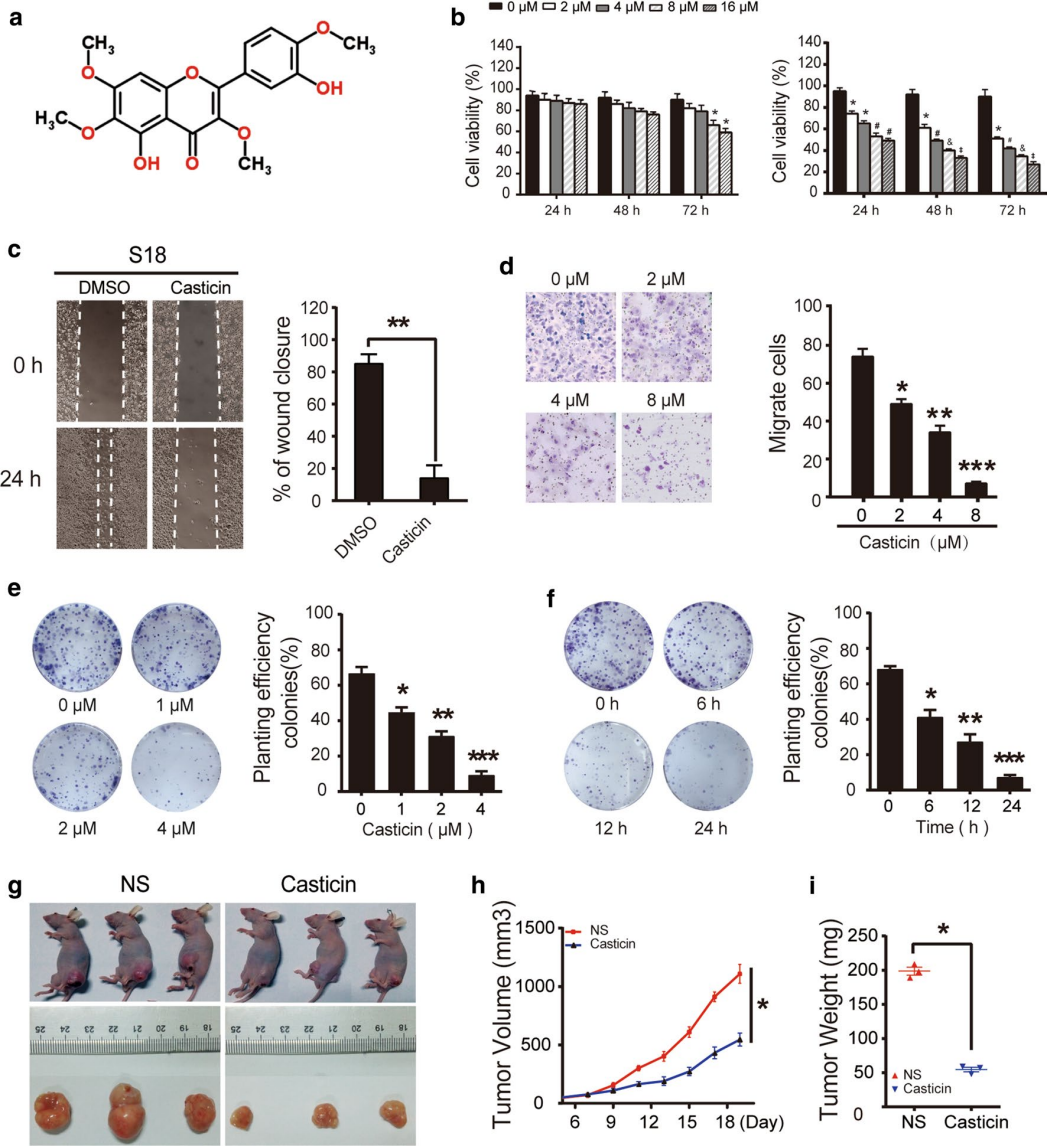
Casticin inhibits the proliferation and viability of NPC cells in vitro and in vivo.** a** The chemical structure of casticin.** b** NP69 and S18 cells were treated with a gradient of casticin concentrations (0, 2, 4, 8, 16 μM) for 24, 48 or 72 h. Cell viability was assessed using the CCK-8 assay. The data are presented as the mean ± SEM, *p< 0.05 versus 0 μM; ^#^p < 0.05 versus 2 μM; ^&^p < 0.05 versus 4 μM; ^‡^p < 0.05 versus 8 μM.** c** Casticin inhibits migration of S18 cells in the wound healing assay. White dashed lines indicate the wound edge. The residual gap between the migrating cells from the opposite edges of the wound is represented by a percentage of the initial scratch area. Corresponding graph shows the mean width of the injury lines of three independent experiments (right). All data are presented as the mean ±standard deviation. **p < 0.01 versus DMSO.** d** Casticin inhibits migration of S18 cells in the Transwell assay. Corresponding graphs in the panel on the right show the mean numbers of cells per high-power field (HPF) from five independent areas. All data are presented as the mean ± standard deviation. *p< 0.05 versus 0 μM, **p < 0.01 versus 2 μM, and ***p< 0.001 versus 4 μM.** e**,** f** Colony formation of S18 cells post-treatment with casticin. Cells were exposed to 0, 1, 2, and 4 μM casticin for 12 h or were exposed to a fixed level of 1 μM casticin for different time periods (0, 6, 12, and 24 h) and were then allowed to form colonies for approximately 10 days. Corresponding graphs show the mean number of colonies in different groups for three independent experiments (right). All data are presented as the mean ± standard deviation. Right panel of** e** *p< 0.05 versus 0 μM, **p< 0.01 versus 1 μM, and ***p< 0.001 versus 2 μM. Right panel of** f** *p< 0.05 versus 0 h, **p< 0.01 versus 6 h, and ***p< 0.001 versus 12 h;** g** Casticin (10% DMSO +90% physiological saline, 40 mg/kg) was injected into nude mice every day for 12 days starting 6 days after inoculation with 5 million S18 cells.** h** Tumour volume was periodically measured for each mouse, and the growth curve was plotted. i Tumours were excised from the animals and weighed. *p < 0.05 versus NS

Also, the legend for Figure 2f needs to read: “Levels of expression of apoptosis related proteins BCL2 and BAX in S18 cells with a gradient of casticin concentrations (0, 1, 2, 4 µM), and C666-1 with a gradient of casticn concentrations (0, 2, 4 or 8 µM) analyzed by Western blotting.”

These changes don’t affect any of the conclusions. The authors apologise for any inconvenience.
