# Supplementary figures and images for: Casticin inhibits nasopharyngeal carcinoma growth by targeting phosphoinositide 3-kinase
Source: Cancer Cell Int. 2019 Dec 21;19:348. doi: 10.1186/s12935-019-1069-6 (PMC6925493; doi:10.1186/s12935-019-1069-6)

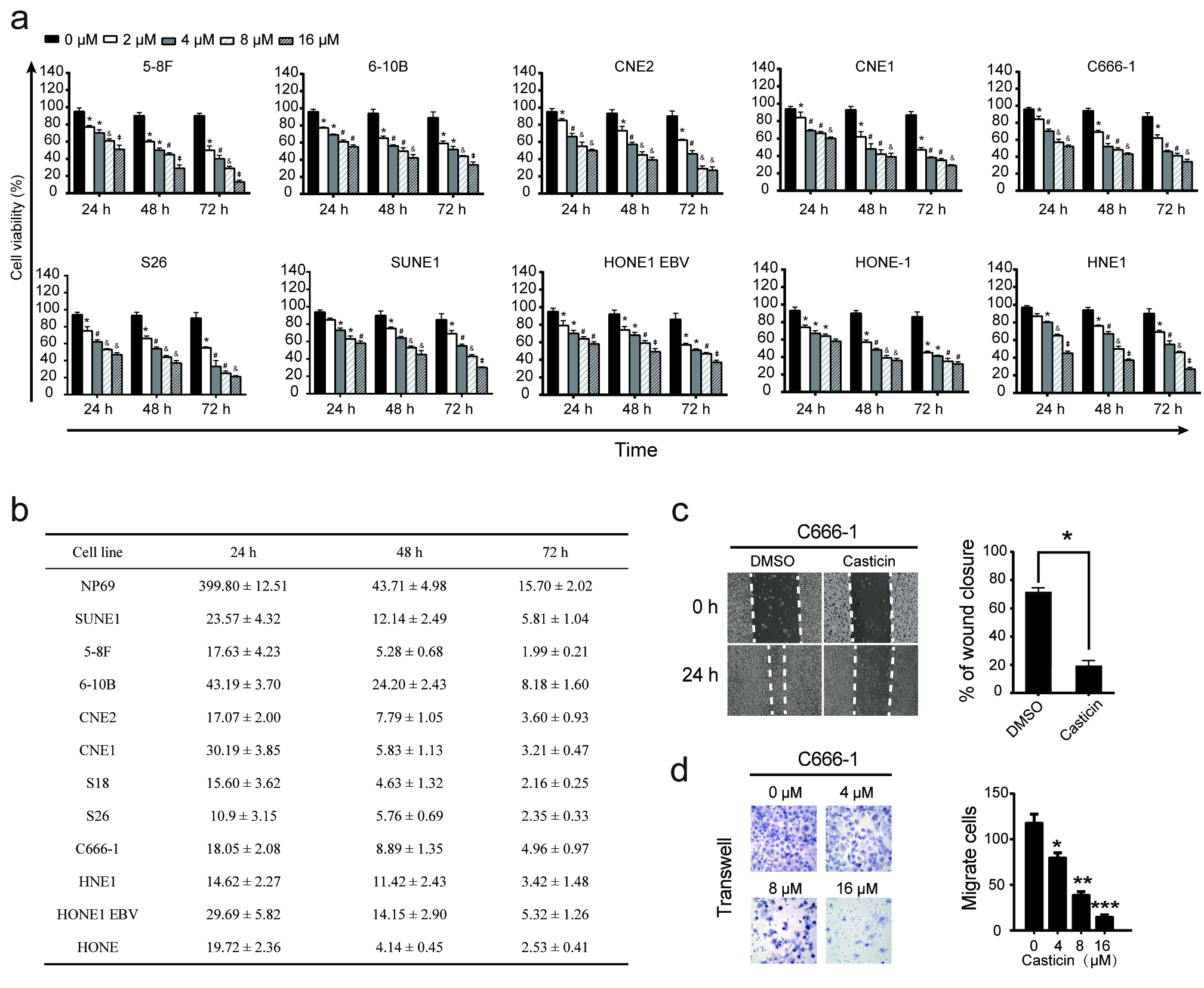

Supplement: Supplementary file 1 — Additional file 1: Fig. S1. Casticin inhibits the viability, migration and invasion of NPC cells. a Ten NPC cell lines were treated with various concentrations of casticin for 24, 48 or 72 h. Cell viability was assessed using the CCK-8 assay. All the data are presented as the mean ± SEM, *p < 0.05 versus 0 µM; #p < 0.05 versus 2 µM; &p < 0.05 versus 4 µM; ‡p < 0.05 versus 8 µM. b IC50 values of casticin in 12 cell lines for 24, 48 or 72 h. c Wound-healing assay of C666-1 cells before and after casticin treatment. White dashed lines indicate the wound edge. The residual gap between the migrating cells from the opposite edges of the wound is represented as a percentage of the initial scratch area. Corresponding graphs show the mean width of the injury lines of three experiments (right). All data are presented as the mean ± standard deviation. *p < 0.01 versus DMSO. d Casticin-induced inhibition of C666-1 migration in the Transwell assay. Corresponding graphs (panel on right) show the mean numbers of cells per high-powerfield (HPF) from five independent areas. All data are presented as the mean ± standard deviation, and the representative experiment shown was repeated three times. *p < 0.05 versus 0 µM, **p < 0.01 versus 4 µM, and ***p < 0.001 versus 8 µM. [file 12935_2019_1069_MOESM1_ESM.tif]

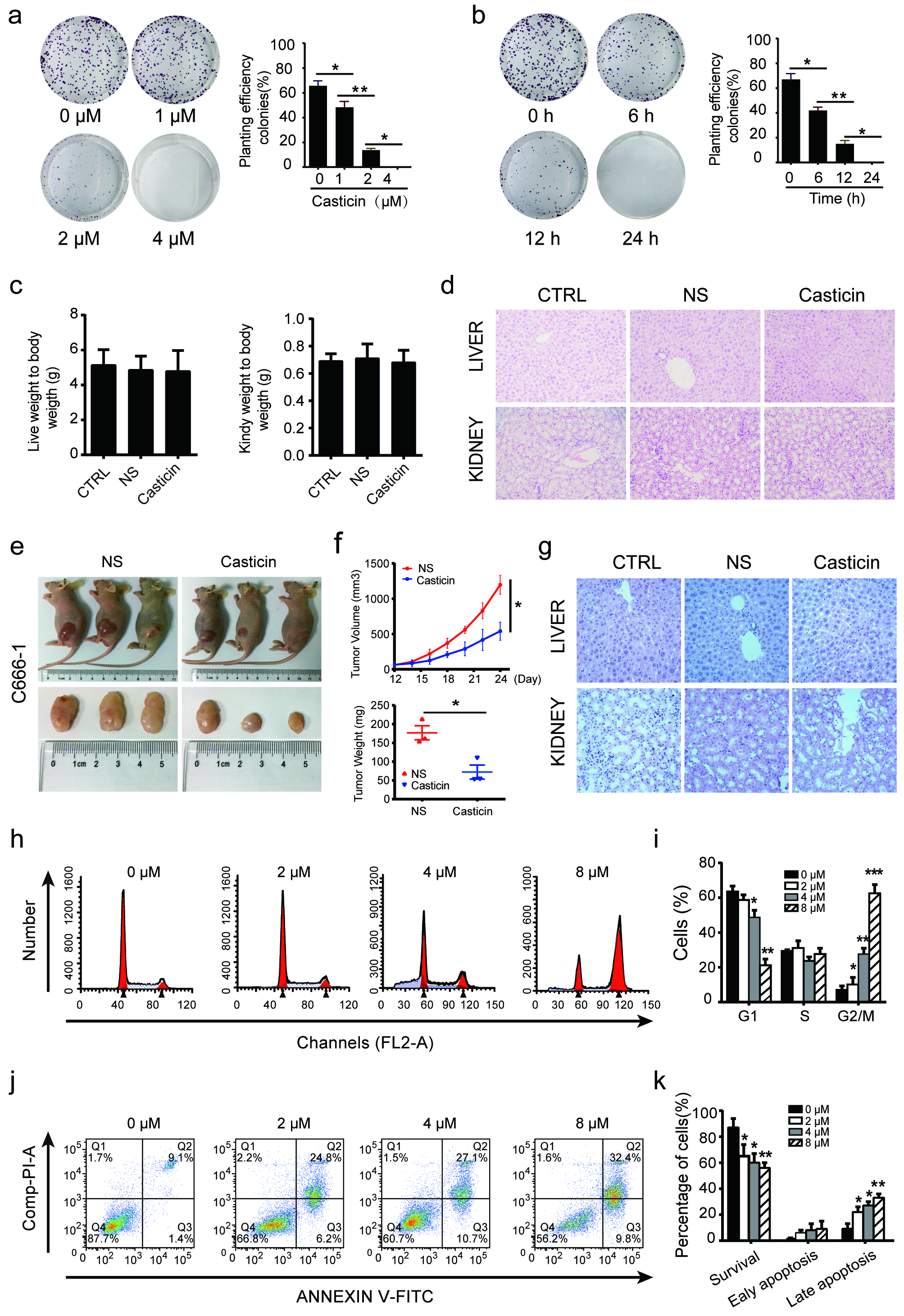

Supplement: Supplementary file 2 — Additional file 2: Fig. S2 Casticin inhibits the proliferation of NPC cells in vitro and in vivo and induces G2/M arrest and apoptosis in the NPC cell line C666-1. a, b Casticin suppressed colony formation of C666-1 cells. Cells were exposed to casticin 0, 1, 2, and 4 µM casticin for 12 h or were treated with 1 µM casticin for different time points (0, 6, 12, and 24 h), and were allowed to form colonies for approximately 10 days. Corresponding graphs show the mean number of colonies formed by different groups for three experiments (right). Right panel of a): *p < 0.05 versus 0 µM, **p < 0.01 versus 2 µM, and ***p < 0.001 versus 4 µM. Right panel of b: *p < 0.05 versus 6 h, **p < 0.01 versus 12 h, and ***p < 0.001 versus 24 h; c Ratios of liver and kidney weight to body weight in three different groups of the S18 nude mouse model; d HE staining of liver and kidney in the experimental group and saline group in regards to the tumorigenicity of the S18 mouse model. e Casticin inhibits tumour growth in vivo. Different concentrations (10% DMSO + 90% physiological saline, 40 mg/kg) of casticin were injected into nude mice once per day after they were inoculated with C666-1 cells. Images of 3 representative mice from each group are presented to show the sizes of the resulting tumours. f Tumour volume was periodically measured for each mouse and tumour growth curves were plotted. Data were used in a parametric generalized linear model with random effects (top of the panel). Tumours were excised from the animals and weighed (bottom of the panel). g HE staining of liver and kidney in the experimental and saline groups campared with the untreated group in the C666-1 nude mouse model. All data are presented as the mean ± standard deviation, and each experiment was repeated three times. *p < 0.05 versus NS. h, i C666-1 cells were treated with casticin at 0, 2, 4 or 8 µM for 24 h. The cell cycle distribution was analysed using flow cytometry; j, k Cell apoptosis was determined by [file 12935_2019_1069_MOESM2_ESM.tif]
